# Morpholino-mediated in vivo silencing of *Cryptosporidium parvum* lactate dehydrogenase decreases oocyst shedding and infectivity

**DOI:** 10.1016/j.ijpara.2018.01.005

**Published:** 2018-07

**Authors:** Xuejin Zhang, Chi Yong Kim, Tori Worthen, William H. Witola

**Affiliations:** Department of Pathobiology, College of Veterinary Medicine, University of Illinois, Urbana-Champaign, USA

**Keywords:** *Cryptosporidium parvum*, Lactate dehydrogenase, In vivo morpholino-based gene knockdown, Mouse infection

## Abstract

*Cryptosporidium* is a highly prevalent protozoan parasite that is the second leading cause of childhood morbidity and mortality due to diarrhoea in developing countries, and causes a serious diarrheal syndrome in calves, lambs and goat kids worldwide. Development of fully effective drugs against *Cryptosporidium* has mainly been hindered by the lack of genetic tools for functional characterization and validation of potential molecular drug targets in the parasite. Herein, we report the development of a morpholino-based in vivo approach for *Cryptosporidium parvum* gene knockdown to facilitate determination of the physiological roles of the parasite’s genes in a murine model. We show that, when administered intraperitoneally at non-toxic doses, morpholinos targeting *C. parvum* lactate dehydrogenase (*Cp*LDH) and sporozoite 60K protein (*Cp*15/60) were able to specifically and sustainably down-regulate the expression of *Cp*LDH and *Cp*15/60 proteins, respectively, in *C. parvum*-infected interferon-γ knockout mice. Over a period of 6 days of daily administration of target morpholinos, *Cp*LDH and *Cp*15/60 proteins were down-regulated by 20- to 50-fold, and 10- to 20-fold, respectively. Knockdown of *Cp*LDH resulted in approximately 80% reduction in oocyst load in the feces of mice, and approximately 70% decrease in infectivity of the sporozoites excysted from the shed oocysts. *Cp*15/60 knockdown did not affect oocyst shedding nor infectivity but, nevertheless, provided a proof-of-principle for the resilience of the morpholino-mediated *C. parvum* gene knockdown system in vivo. Together, our findings provide a genetic tool for deciphering the physiological roles of *C. parvum* genes in vivo, and validate *Cp*LDH as an essential gene for the growth and viability of *C. parvum* in vivo.

## Highlights

• An in vivo morpholino-based approach for targeted gene knockdown of genes in *Cryptosporidium parvum* was developed.
• *Cryptosporidium parvum* lactate dehydrogenase, and sporozoite 60K were knocked down sustainably in infected mice.
• *Cryptosporidium parvum* lactate dehydrogenase knockdown significantly decreased oocyst shedding and infectivity.

## Graphical abstract

**Figure:**
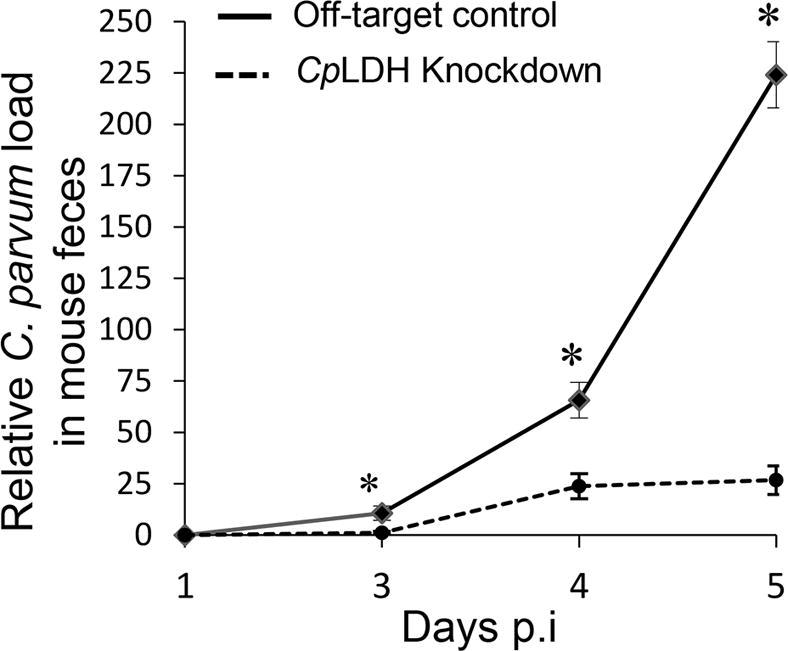

## 1 Introduction

*Cryptosporidium* spp. (*Cryptosporidium parvum* and *Cryptosporidium hominis*) are highly prevalent protozoan parasites that rank as the second leading cause of childhood morbidity and mortality due to diarrhoea in developing countries (Kotloff et al., 2013, Checkley et al., 2015). In livestock, *C. parvum* infection results in a serious diarrheal illness with significant production losses in calves, lambs and goat kids (De Graaf et al., 1999, Jex and Gasser, 2009, Karanis et al., 2010). Efforts to develop fully effective drugs for treating *Cryptosporidium* infections have largely been hampered by the lack of genetic tools for interrogating and validating potential drug molecular targets in the parasite during the various life cycle stages.

In efforts aimed at developing genetic tools for studying *Cryptosporidium*, a clustered regularly interspaced short palindromic repeat (CRISPR)/Cas9 gene knockout system for *Cryptosporidium* was reported (Vinayak et al., 2015). We have also recently reported an antisense approach for an in vitro targeted gene knockdown system in *C. parvum* using phosphorodiamidate morpholino oligomers (morpholinos) (Witola et al., 2017). However, *Cryptosporidium* can only be cultured transiently in vitro (Upton et al., 1994), and this does not allow for development of all the life cycle stages of the parasite. Nevertheless, *Cryptosporidium* can be continuously propagated in experimental murine models in which it completes its life cycle. The life cycle of *C. parvum* in murine models starts with the excystation of sporozoites from ingested oocysts in the gastrointestinal tract of the host. The sporozoites invade host enterocytes and transform into merozoites which then multiply asexually by merogony, with the resulting merozoites infecting more host enterocytes. The sexual stage (gametogony) follows the formation of second-generation merozoites that invade other enterocytes to form numerous macrogamonts and microgamonts. Fertilisation of the macrogamonts by microgamonts results in the formation of the zygote-containing oocysts that sporulate inside the host and are passed in the host animal’s faeces (Current and Reese, 1986, Uni et al., 1987).

In the present study, we endeavoured to develop a morpholino-based in vivo assay for *C. parvum* gene knockdown in a murine model that would facilitate interrogation of gene function during the various life cycle stages of the parasite. Morpholinos are synthetic chains of six-membered non-ionic DNA analogues that block RNA splicing or initiation of protein translation by Watson/Crick base-pairing with target mRNA (Moulton et al., 2004, Morcos, 2007). When administered parenterally, morpholinos conjugated to a delivery moiety are able to enter a wide variety of tissues in living mice (Morcos et al., 2008), and have been shown to effectively knockdown targeted genes in various tissue cells (Ferguson et al., 2013). Herein, we report that morpholinos used at non-toxic doses are able to sustainably knockdown targeted *C. parvum* genes in interferon-γ knockout mice infected with the parasite. By targeting the *C. parvum* lactate dehydrogenase gene (*Cp*LDH), we provide genetic evidence that the *Cp*LDH gene is essential for the parasite’s survival, growth and viability in vivo.

## 2 Materials and methods

### 2.1 *Cryptosporidium parvum* strain propagation and purification of oocysts

The AUCP-1 isolate of *C. parvum* was maintained and propagated in male Holstein calves in accordance with the guidelines of protocol number 15123 approved by the University of Illinois at Urbana-Champaign, USA. Freshly shed *C. parvum* oocysts in calf faeces were extracted and purified by sequential sieve filtration, Sheather's sugar flotation, and discontinuous sucrose density gradient centrifugation, essentially as previously described (Arrowood and Sterling, 1987, Current, 1990). The purified oocysts were rinsed and stored at 4 °C in 50 mM Tris, 10 mM EDTA, pH 7.2, and used within 1 to 2 weeks during which time viability remained above 80% as determined by excystation.

### 2.2 Design of morpholino oligomers

Morpholinos specific for inhibiting the translation of *Cp*LDH and *C. parvum* sporozoite 60K protein (*Cp*15/60) mRNA were designed as previously described (Witola et al., 2017). Briefly, each morpholino was designed as a 25 base sequence including the start codon and downstream bases that would specifically bind to its complementary target mRNA site via Watson–Crick pairing, and sterically block the translation initiation complex. The morpholino sequences were based on mRNA sequences reported in the CryptoDB database with identification numbers Cgd7_480-t26_1 and Cgd7_4540-t26_1, for *Cp*LDH and *Cp*15/60, respectively. Specifically, the *Cp*LDH target morpholino sequence was 5′-CGGCAATCTTGCGTCTTTCAATCAT-′3 (with the start codon underlined), while the *Cp*15/60 target morpholino sequence was 5′-CAACAGGATTTCAAGTTACCCATGT -3′ (with the start codon underlined). An off-target standard control morpholino sequence was 5′-CCTCTTACCTCAGTTACAATTTAT-3′. The morpholinos were synthesised with an octa-guanidine dendrimer delivery moiety covalently linked at the 3′-end and named vivo morpholinos (Gene Tools, USA).

### 2.3 Mouse infection and *C. parvum* gene knockdown assays

Gamma interferon knockout mice (B6.129S7-Ifng), 6 weeks of age, were purchased from Charles River, USA. The care and use of the mice was done following the guidelines of protocol number 17024 approved by the University of Illinois at Urbana-Champaign, USA. The animals were allowed to acclimatise for 1 week before experiments commenced. Morpholinos were reconstituted in sterile distilled water. Prior to commencement of the knockdown assays in mice, the tolerability of each morpholino was tested by i.p. administration of doses of 0.00, 6.25, 12.50, 18.75 and 25.00 nmoles to groups of mice (three mice for each dose) daily for 7 days. Mice were observed daily for signs of toxicity including changes from normal physical activity, respiration, body temperature, feeding, posture, fur condition or occurrence of death.

For target gene knockdown assays, for each morpholino, mice were allocated to two treatment groups of ‘Infected plus target morpholino’, and ‘Infected plus off-target morpholino’, with each group having three mice. Starting 1 day prior to infection, mice in each group received 12.5 nmoles i.p. injection of their respective morpholino per 20 g body weight. On day 2 of treatment, each mouse received 20,000 oocysts of *C. parvum* in 50 µl of PBS by oral gavage. Mice were housed individually, and on day 2 p.i. the bedding in the mouse cages was removed and cage bottoms lined with sterile gauze. Starting at day 3 p.i., faecal pellets were collected daily from each cage and placed in individual sterile 15 ml conical tubes. An equivalent volume of PBS containing a cocktail of penicillin (100 units/ml), streptomycin (100 µg/ml), chloramphenicol (34 µg/ml) and amphotericin (0.25 µg/ml) was added to the faecal samples and stored at 4 °C until use. Three independent replicate infection assays were performed.

### 2.4 *Cryptosporidium parvum* oocysts purification from mouse faeces

*Cryptosporidium parvum* oocysts shed by mice were purified from the faecal samples following the method of von Oettingen et al. (2008), with some modifications. Briefly, for each mouse 3 g of faecal sample was homogenised in 7 ml of ice-cold distilled sterile water with a spatula followed by vortexing, to make a fine slurry, and the volume made up to 14 ml with water. The faecal suspension was spun down at 2000*g* for 10 min at 4 °C and the supernatant discarded. The pellet was resuspended in 2.5 ml of water, the volume made up to 25 ml with ice-cold saturated sodium chloride solution and thoroughly mixed by vortex. The suspension was carefully overlaid with 5 ml of ice-cold water and centrifuged at 2000*g* for 15 min at 4 °C. Approximately 7.5 ml were carefully aspirated from the interphase using a pipette and transferred to a 50 ml conical tube. The extraction process was repeated twice more by mixing the remaining suspension and overlying it with 5 ml of ice-cold water followed by centrifugation and collection of 7.5 ml from the interface. For each sample, the interface aspirates were pooled in a 50 ml conical tube and the volume made up to 50 ml with ice-cold water followed by centrifugation at 2000*g* for 15 min at 4 °C. The supernatant was removed and the oocysts contained in the pellet were washed twice by centrifugation with 10 ml of ice-cold water. The final pellet was resuspended in 100 µl of ice-cold PBS containing a cocktail of penicillin/streptomycin/amphotericin, and enumerated by hemocytometer. The oocysts were preserved at 4 °C until use.

### 2.5 Western blot analysis of effect of morpholino on target gene expression in *C. parvum*

To determine the knockdown effects of morpholinos on target genes in *C. parvum*, 10^5^ oocysts extracted from the off-target morpholino-treated and the target morpholino-treated mice were suspended in uniform volumes of PBS, lysed in laemmli sample buffer and boiled for 5 min. Equal amounts of the samples were fractionated by SDS–PAGE and transferred onto nitrocellulose membranes. Immunoblotting was performed using mono-specific purified rat anti-*Cp*LDH antibodies (Witola et al., 2017) or *Cp*15/60 sporozoite 60K protein monoclonal antibody (LifeSpan Biosciences, Inc., USA) at 1:200 dilution as primary antibodies. Horseradish peroxidase (HRP)-conjugated chicken anti-rat (ThermoFisher Scientific, USA) and HRP-goat anti-mouse (ThermoFisher Scientific, USA) were used as secondary antibodies at 1:2000 dilution. Signal generation was performed using Clarity Western ECL Substrate (Bio-Rad, USA) and imaging done using the FluoroChem R imager (Protein Simple, USA).

### 2.6 Analysis of effect of gene knockdown on infectivity of excysted *C. parvum* sporozoites

Sporozoites were excysted from equal amounts of *C. parvum* oocysts extracted from faecal samples of mice as described in Section 2.4, following the method described previously (Kuhlenschmidt et al. 2016). Briefly, for each sample, 5 × 10^4^ purified *C. parvum* oocysts were suspended in 500 µl of PBS and an equal volume of 40% commercial laundry bleach added and incubated for 10 min at 4 °C. This was followed by four washes in PBS containing 1% (w/v) BSA. The oocysts were then resuspended in Hanks balanced salt solution (HBSS) and incubated at 37 °C for 60 min. Following incubation, the oocysts were mixed with an equal volume of warm 1.5% sodium taurocholate in HBSS and incubated for a further 60 min at 37 °C to excyst sporozoites. The excysted sporozoites were collected by centrifugation, suspended in RPMI 1640 medium supplemented with sodium bicarbonate (2 g/L), glucose (2.5 g/L), 10% foetal bovine serum (Gibco), 1× antibiotic–antimycotic (Gibco), and 1× sodium pyruvate (Gibco). The sporozoites were purified from oocyst shells by passing the suspension through a sterile 5 µM syringe filter (Millex, USA), followed by enumeration by hemocytometer.

For the infection assay, to freshly confluent human colorectal tumour (HCT-8) cells cultured in 96-well plates, 200 µl of fresh supplemented-RPMI medium were added. Then, 10^4^ of the freshly excysted sporozoites were added to each well and the plates incubated at 37 °C with 5% CO_2_ for 48 h or 72 h. Following incubation, the cultures were processed for immunofluorescence analysis as described previously (Kuhlenschmidt et al., 2016). Briefly, the medium was decanted from culture wells, and the cells fixed with methanol-acetic acid (9:1) for 2 min at room temperature. The cells were permeabilized by two successive washes with wash buffer (0.1% Triton X-100, 0.35 M NaCl, 0.13 M Tris-base, pH 7.6), blocked with 5% normal goat serum, and stained with antibody to *C. parvum* (SporoGlo; Waterborn, Inc., USA) overnight at 4 °C. The stained cells were washed twice with PBS followed by water, and observed with an inverted fluorescence microscope with a 40× objective. Fluorescence quantification was done using ImageJ version 1.37v software (National Institutes of Health, USA).

### 2.7 Real-time PCR quantification of *C. parvum* load in faecal samples

Faecal samples collected from mice infected with *C. parvum* and treated with morpholinos as described in Section 2.3 were used for extraction of genomic DNA. For each homogenised faecal sample, 220 mg were used to extract genomic DNA with the QIAamp DNA Stool Mini Kit (Qiagen, USA) following the manufacturer’s instructions. Quantification of the amount of *C. parvum* 18s rRNA gene (GenBank accession number AF164102) was done following the method of Parr et al. (2007), with some modifications. The primer pair used was 5′-CTGCGAATGGCTCATTATAACA-3′ (Forward), and 5′-AGGCCAATACCCTACCGTCT-3′ (Reverse). This primer pair generated a DNA fragment of 240 bp from *C. parvum* genomic DNA by conventional PCR. The 240 bp PCR product was fractionated on agarose gel, extracted using the QIAquick® Gel extraction kit (Qiagen, USA), and the concentration measured by Nanodrop Spectrophotometer (Fisher, USA). Ten-fold serial dilutions of the extracted DNA fragment were made and used as quantification standards for real-time PCR. Each real time PCR mixture contained 2 μl of DNA template, 1 μl of primer mix (500 nM (each)), and 10 μl of SsoAdvanced Universal SYBR Green Supermix (Bio-Rad, USA), with the final volume made up to 20 μl with nuclease-free water. The cycling conditions included an initial denaturation for 10 min at 98 °C, 40 cycles at 98 °C for 15 s and 60 °C for 1 min, and a final melting curve step. Cycling was performed using a 7500 Real Time PCR System (Applied Biosystems, USA). DNA quantities were derived by the system software using the generated quantification standard curves.

### 2.8 Statistical analyses

Statistical analyses were performed using two-tailed Student's *t* test. *P* values of 0.05 or less were considered significant

## 3 Results

### 3.1 Determination of tolerable dose levels of morpholino in mice

Gene Tools LLC (USA) has optimised morpholino use of up to 25 nmoles for a typical 20 g black mouse (4–10 weeks old) daily by i.v. or i.p. injection. For all the morpholinos (*Cp*LDH, *Cp*15/60 and off-target) tested, 12.50 nmoles per 20 g of mouse body weight was found to be the highest dose that consistently did not induce any of the toxicity signs (changes from normal physical activity, respiration, body temperature, feeding pattern, body posture, fur condition or occurrence of death) over the 7 day period of administration in mice. The dose of 12.50 nmoles per 20 g of mouse body weight was, therefore, used as the optimum dose in the subsequent assays.

### 3.2 *Cp*LDH and *Cp*15/60 down-regulate expression of target proteins in *C. parvum* in vivo

**Fig. 1:**
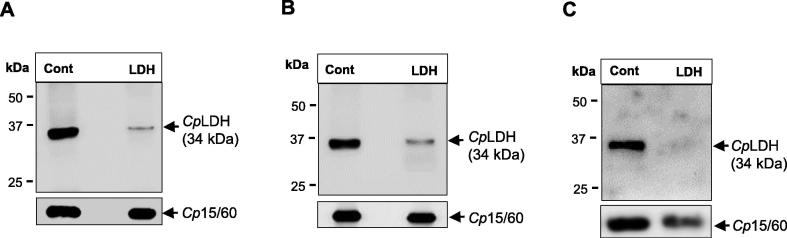
Western blot analysis of the in vivo knockdown of *Cryptosporidium parvum* lactate dehydrogenase (*Cp*LDH) by morpholino. *Cryptosporidium parvum-*infected mice were injected daily with either off-target or *Cp*LDH-target morpholino. Equal amounts of *C. parvum* oocysts isolated from the faeces of mice at (A) 3 days p.i., (B) 4 days p.i. and (C) 6 days p.i. were lysed in Laemmli buffer and equal protein lysates were analysed for the expression of *Cp*LDH protein. Lane Cont: protein lysate of oocysts from off-target morpholino-treated mice; lane LDH: protein lysate of oocysts from *Cp*LDH-target morpholino-treated mice. As a loading control, panels (*Cp*15/60) show equal amounts of the protein lysates blotted using antibody against *C. parvum Cp*15/60 sporozoite protein. The data shown is representative of three biological replicates for each treatment group. Three independent replicate experiments are represented.

**Fig. 2:**
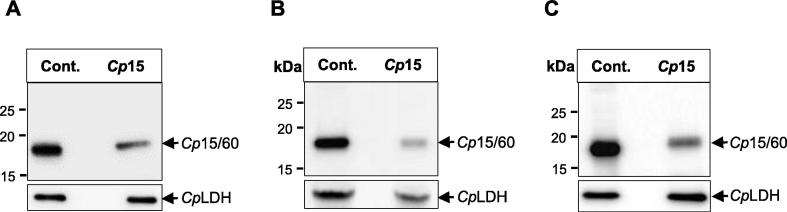
Western blot analysis of the in vivo knockdown of *Cryptosporidium parvum Cp*15/60 sporozoite protein by morpholino. *Cryptosporidium parvum-*infected mice were injected daily with either off-target or *Cp*15/60-target morpholino. Equal amounts of *C. parvum* oocysts isolated from the faeces of mice at (A) 3 days p.i., (B) 4 days p.i. and (C) 6 days p.i. were lysed in Laemmli buffer and equal protein lysates analysed for the expression of *Cp*15/60 protein. Lane Cont: protein lysate of oocysts from off-target morpholino-treated mice; lane *Cp*1*5*: protein lysate of oocysts from *Cp*15/60-target morpholino-treated mice. As a loading control, panels (*Cp*LDH) show equal amounts of the protein lysates blotted using antibody against *C. parvum* lactate dehydrogenase (LDH) protein. The data shown is representative of three biological replicates for each treatment group. Three independent replicate experiments are represented.

To assess the knockdown effect of *Cp*LDH- and *Cp*15/60-target morpholinos on the expression of their respective target proteins in *C. parvum* during infection of mice, equal amounts of oocysts purified from the mouse faeces on different days of treatment were analysed by western blot. One day prior to infection of the mice with *C. parvum*, morpholino administration was started to facilitate advance distribution of the morpholino to mouse tissues before the infection could be established. Starting from day 3 p.i., oocysts were detectable in the faecal samples, and therefore, we elected to start the analysis from this day. Both *Cp*LDH- and *Cp*15/60-target morpholinos were found to significantly reduce the expression of their target proteins in the oocysts collected from faeces at all the time points (3, 4, and 6 days p.i.) analysed compared with the off-target morpholino (Fig. 1, Fig. 2). By densitometric analysis of the western blot protein bands, the *Cp*LDH-target morpholino reduced the *Cp*LDH protein amount by 30-fold (±5.0), 20-fold (±2.0) and 50-fold (±7.0) at 3, 4 and 6 days p.i., respectively. *Cp*15/60-target morpholino decreased the amount of *Cp*15/60 protein by 15-fold (±1.5), 10-fold (±0.8) and 20-fold (±1.3) at 3, 4 and 6 days p.i., respectively. It is noteworthy that, by visual analysis, the fold-change reduction in protein band intensities for both *Cp*LDH and *Cp*15/60 due to morpholino treatment may be more than that obtained by densitometric analysis because the control bands appeared to be saturated. For mice treated with *Cp*LDH-target morpholino, western blotting was done on equivalent amounts of the oocysts’ protein lysates using an antibody against *Cp*15/60 protein as the control for protein loading, and to determine the specificity of the morpholino. As shown in Fig. 1, *Cp*LDH-target morpholino did not decrease the expression of *Cp*15/60 protein compared with the effect it had on *Cp*LDH protein. Similarly, the *Cp*15/60-target morpholino did not alter the expression of *Cp*LDH protein compared with its effect on the *Cp*15/60 protein (Fig. 2). These findings indicated that the two morpholinos down-regulated the expression of their specific target proteins.

### 3.3 *Cp*LDH knockdown decreases *C. parvum* oocyst shedding in mouse faeces

**Fig. 3:**
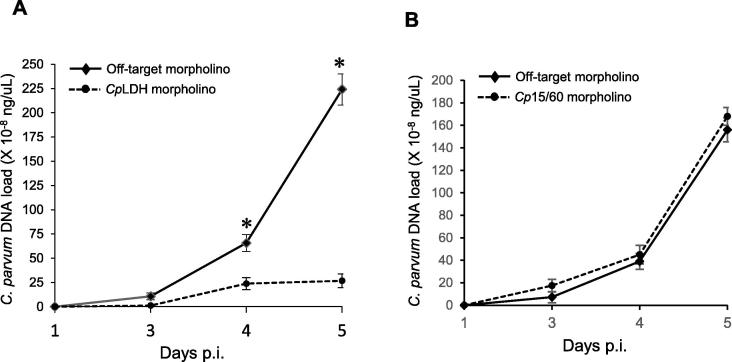
Real-time PCR analysis of the load of *Cryptosporidium parvum* DNA in faecal samples of mice treated with morpholinos and infected with *C. parvum*. (A) *Cryptosporidium parvum-*infected mice were injected daily with either off-target morpholino or *C. parvum* lactate dehydrogenase (*Cp*LDH)-target morpholino. Genomic DNA was extracted from equal amounts of mouse faecal samples collected on various days p.i., and the concentration of the *C. parvum* 18 s rRNA gene fragment was quantified. Solid black line depicts *C. parvum* DNA quantity in faecal samples from mice treated with off-target morpholino. Dashed line shows *C. parvum* DNA quantity in faecal samples from mice treated with *Cp*LDH-target morpholino. (B) *Cryptosporidium parvum-*infected mice were injected daily with either off-target or *C. parvum Cp*15/60 sporozoite protein mRNA (*Cp*15/60)-target morpholino. Genomic DNA was extracted from equal amounts of mouse faecal samples collected at different days p.i., and the load of the *C. parvum* 18 s rRNA gene fragment was quantified. Solid black line depicts DNA quantity in faecal samples from mice treated with off-target morpholino. Dashed line shows DNA quantity in faecal samples from mice treated with *Cp*15/60-target morpholino. The data shown represent means for three independent experiments with standard error bars and levels of statistical significance depicted (^*^P < 0.05).

Having ascertained that both *Cp*LDH- and *Cp*15/60 target morpholinos consistently and effectively down-regulated their target proteins at all time points tested, we endeavoured to determine their effect on oocyst shedding in mouse faeces. Equal amounts of homogenised faecal samples were used to extract genomic DNA and then equivalent volumes of the extracted DNA solutions were used as templates for real-time PCR quantification of the *C. parvum* 18 s rRNA gene fragment. As shown in Fig. 3A, while the *C. parvum* DNA load exponentially increased in mice treated with off-target morpholino, it stayed consistently low in the faeces of mice treated with the *Cp*LDH-target morpholino. These findings indicated that *Cp*LDH knockdown reduced the shedding of *C. parvum* oocysts in the faeces of treated mice. In contrast, knockdown of *Cp*15/60 protein did not significantly affect the shedding of *C. parvum* oocysts in the *Cp*15/60-target morpholino-treated mice compared with those treated with the off-target morpholino (Fig. 3B).

### 3.4 *Cp*LDH knockdown reduces infectivity of *C. parvum* sporozoites

**Fig. 4:**
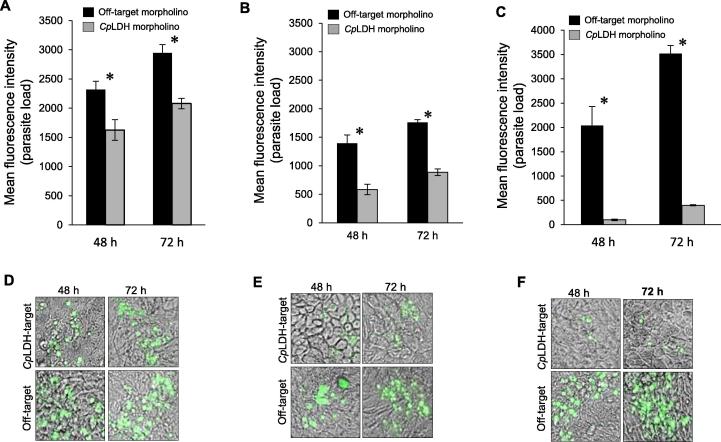
Analysis of the infectivity of *Cryptosporidium parvum* sporozoites excysted from oocysts shed by mice treated with off-target or *Cp*LDH-target morpholinos. Equal amounts of freshly excysted sporozoites from off-target morpholino-treated (black columns) and *Cp*LDH-target morpholino-treated (grey columns) mice at (A) 4 days p.i., (B) 5 days p.i. and (C) 6 days p.i. were inoculated into human colorectal tumour (HCT-8) cells in culture and analysed for infectivity and proliferation by an immunofluorescence assay after 48 h and 72 h of culture. The fluorescence generated by intracellular *C. parvum* merozoites was quantified and is shown on the *Y*-axis representing the relative parasite load. Representative images of immunofluorescence staining of the HCT-8 cells infected with sporozoites from mice after (D) 4 days p.i., (E) 5 days p.i. and (F) 6 days p.i. Green fluorescence depicts intracellular *C. parvum* merozoites (×40 objective). The data shown in A–C represent means of three independent experiments with standard error bars and levels of statistical significance between groups indicated (^*^P < 0.05).

**Fig. 5:**
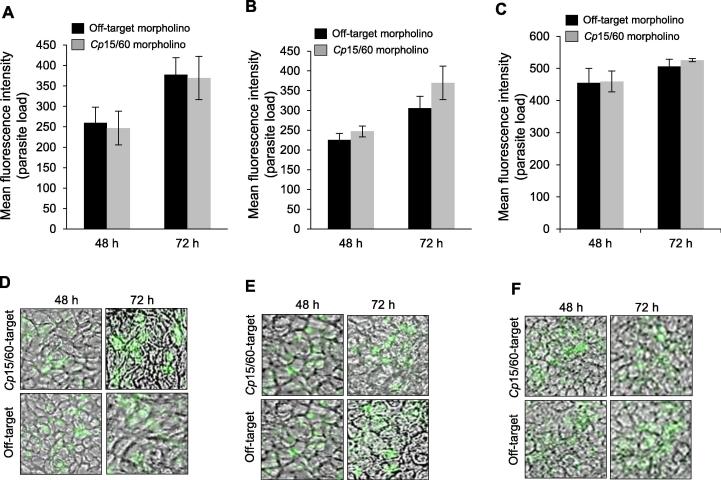
Analysis of the infectivity of *Cryptosporidium parvum* sporozoites exysted from oocysts shed by mice treated with off-target or *Cp*15/60-target morpholinos. Equal amounts of freshly exysted sporozoites from off-target morpholino-treated (black columns) and *Cp*15/60-target morpholino-treated (grey columns) mice at (A) 4 days p.i., (B) 5 days p.i. and (C) 6 days p.i. were inoculated into human colorectal tumour (HCT-8) cells in culture and analysed for infectivity and proliferation after 48 h and 72 h of culture by an immunofluorescence assay. The green fluorescence generated by intracellular *C. parvum* merozoites was quantified and is shown on the *Y*-axis representing the relative parasite load. Representative images of immunofluorescence staining of the HCT-8 cells infected with sporozoites from mice after (D) 4 days p.i., (E) 5 days p.i. and (F) 6 days p.i. Green fluorescence depicts intracellular *C. parvum* merozoites (×40 objective). The data shown in A-C represent means of three independent experiments with standard error bars.

To assess the effect of *Cp*LDH or *Cp*15/60 knockdown on the viability of the shed oocysts, we excysted sporozoites from the oocysts that were purified from faecal samples collected from mice at 4 days, 5 days and 6 days p.i. with *Cp*LDH-, *Cp*15/60- or off-target morpholino treatment, and used them to infect HCT-8 cells in vitro. By immunofluorescence assays, we analysed the infection of HCT-8 cells with *C. parvum* and quantified the growth of *C. parvum* in the infected HCT-8 cells at 48 h and 72 h p.i. While cultures infected with sporozoites from mice treated with the off-target morpholino showed a progressive increase in the amount and size of the parasite plaques, cultures infected with sporozoites from mice treated with the *Cp*LDH-target morpholino had significantly fewer parasites with smaller plaque sizes (Fig. 4A–F). Notably, the infectivity of the sporozoites appeared to decrease proportionate to the increase in the number of days the mice were treated with the *Cp*LDH morpholino prior to collection of faecal samples (Fig. 4A–C). This suggested that *Cp*LDH knockdown had a cumulative effect on the viability of the shed oocysts over time. On the other hand, even though treatment of mice with the *Cp*15/60-target morpholino resulted in significant knockdown of *Cp*15/60 protein expression, there was no significant difference in the growth of parasites between the off-target morpholino-treated and the *Cp*15/60-target morpholino-treated parasites at both 48 h and 72 h p.i. of HCT-8 cells in vitro (Fig. 5). This suggested that, unlike *Cp*LDH, *Cp*15/60 does not play a role in the viability and infectivity of *C. parvum* oocysts.

## 4 Discussion

The complete and annotated genome sequence of *Cryptosporidium* indicates that while the parasite lacks conventional molecular drug targets found in other important apicomplexan parasites, it has several potentially targetable plant-like and bacteria-like enzymes (Abrahamsen et al., 2004). To be validated, these potential targets will have to be functionally characterised using genetic tools amenable to *Cryptosporidium*, but that are presently extremely limited. We recently adapted the use of morpholinos for targeted gene knockdown in *C. parvum* in vitro (Witola et al., 2017). However, despite recent reports on the development of engineered cell culture systems for in vitro continuous culture of *C. parvum* (Morada et al., 2016, DeCicco RePass et al., 2017), there is still no validated in vitro culture system that can facilitate the development of *Cryptosporidium* through its various life cycle stages. As such, it remains a challenge to decipher the functions of target genes through the various life stages of the parasite using in vitro culture systems. Therefore, in the present study, we endeavoured to develop a morpholino-based gene knockdown system for *Cryptosporidium* in an interferon-γ knockout mouse model that would facilitate the functional analysis of target genes through the complete life cycle of the parasite. The interferon-γ knockout mouse model can easily be infected with *C. parvum*, leading to clinical disease, with completion of the parasite life cycle and shedding of oocysts in the mouse faeces (Griffiths et al., 1998).

The morpholinos we used in the present study are termed “Vivo morpholinos” because they are designed bearing an octa-guanidine dendrimer delivery moiety covalently linked at the 3′-end, which facilitates their transport into cells by endocytosis, and are protected from protease and nuclease degradation, making them stable (Li and Morcos, 2008, Morcos et al., 2008). They have been successfully used for protein knockdown in various tissues of mice using i.v. and i.p. routes of administration with no adverse toxic or immunogenic side-effects (Morcos et al., 2008, Wu et al., 2009, Nazmi et al., 2010, Wu et al., 2011, Chen et al., 2016).

By targeting the *Cp*LDH and *Cp*15/60 transcripts individually, we attained sustained knockdown of both *Cp*LDH and *Cp*15/60 protein expression over the several days that the morpholinos were administered to the mice at non-toxic doses. We have previously used the same *Cp*LDH-target morpholino and found it to effectively knockdown *Cp*LDH protein during transient culture of *C. parvum* in vitro (Witola et al., 2017), which attests to the consistency, robustness and versatility of the morpholino-based gene knockdown system in *C. parvum*. We found that knockdown of *Cp*LDH in vivo significantly reduced the amount of *C. parvum* oocysts shed by the mice, and that sporozoites excysted from those oocysts had significantly reduced infectivity and proliferative ability in HCT-8 cells in vitro. This implied that not only is *Cp*LDH essential for growth and propagation of *C. parvum*, it is also important for viability of the shed oocysts.

The *Cp*LDH protein in *C. parvum* is a bacteria-type lactate dehydrogenase enzyme that the parasite uses to generate metabolic energy (ATP) in the glycolytic pathway (Madern et al., 2004, Zhang et al., 2015), since *C. parvum* lacks both the Krebs cycle and the cytochrome-based respiration chain (Abrahamsen et al., 2004). In extracellular sporozoites and merozoites, *Cp*LDH has been shown to be localised in the cytosol (Zhang et al., 2015), implying that it is important for generation of parasite energy during these stages and would, therefore, be important during the host cell invasion process. Indeed, proteomic and genomic analyses of *Cryptosporidium* have indicated that glycolysis is the sole energy source in *Cryptosporidium* (Entrala and Mascaró, 1997, Xu et al., 2004, Siddiki, 2013), which is consistent with our findings that *Cp*LDH is essential for growth, propagation and viability of *C. parvum* in vivo.

While the *Cp*15/60-target morpholino significantly down-regulated the expression of *Cp*15/60 protein compared with the off-target morpholino, it did not significantly affect the shedding of oocysts, nor did it alter the in vitro infectivity of the excysted sporozoites. This is consistent with *Cp*15/60 not being essential for growth and viability of *C. parvum*. Nevertheless, the successful morpholino-based knockdown of *Cp*15/60 and *Cp*LDH in vivo provided a proof-of-principle and attested to the versatility of this system in *Cryptosporidium*.

In conclusion, in this study we developed an approach for in vivo functional characterization and validation of *Cryptosporidium* genes. Further, we genetically validated that *Cp*LDH is essential for *C. parvum* growth, oocyst shedding as well as viability and infectivity of the shed oocysts. Corroboratively, previous studies have shown that a *Cp*LDH inhibitor, gossypol, decreases the growth of *Cryptosporidium* in vitro (Zhang et al., 2015). Therefore, considering that *Cp*LDH is unique to *Cryptosporidium*, and very different from the lactate dehydrogenase enzyme found in mammals (Madern et al., 2004), our findings validate *Cp*LDH as a molecular target for the development of effective anti-*Cryptosporidium* drugs.
